# Age of Religious Newspapers

**Published:** 1863-04

**Authors:** 


### AGE OF RELIGIOUS NEWSPAPERS.

1. *The Herald of Gospel Liberty* was first published at Portsmouth, New Hampshire, in September, eighteen hundred and eight.

2. *The Religious Remembrancer* was first published at Philadelphia, September the fourth, eighteen hundred and thirteen, by J. W. Scott, an elder in the Presbyterian Church, and which continued six years.

3. *The Boston Recorder* was first published at Boston, Massachusetts, on the third day of January, eighteen hundred and sixteen, by Nathaniel Willis, now living.

4. *The Religious Intelligencer* was first published at New Haven, Connecticut, on the first day of June, eighteen hundred and sixteen, then under the care of Deacon Nathan Whiting.

5. *The Congregational Journal* was first issued, although under a different name, on the third day of January, eighteen hundred and nineteen.

6. *The Christian Watchman* (now the Christian Watchman and Reflector) was first issued at Boston, Massachusetts, on the twenty-ninth of May, eighteen hundred and nineteen.

### RECAPITULATION.

| *Name.* | *Place.* | *Date.* |
|---|---|---|
| Herald of Gospel Liberty, | Portsmouth, N. H., | Sept. 1808. |
| Religious Remembrancer, | Philadelphia, Pa., | Sept. 4, 1813. |
| Boston Recorder, | Boston, Mass., | Jan. 3, 1816. |
| Religious Intelligencer, | New Haven, Conn., | June 1, 1816. |
| Congregational Journal, | Boston, Mass., | Jan. 3, 1819. |
| Christian Watchman, | Boston, Mass., | Jan. 29, 1819 |

